# Determination of Optimal Doses and Minimum Effective Concentrations of Tricaine Methanesulfonate, 2-Phenoxyethanol and Eugenol for Laboratory Managements in Nile Tilapia (*Oreochromis niloticus*)

**DOI:** 10.3390/ani11061521

**Published:** 2021-05-24

**Authors:** Tirawat Rairat, Yu Chi, Chia-Yu Hsieh, Yi-Kai Liu, Niti Chuchird, Chi-Chung Chou

**Affiliations:** 1 Department of Fishery Biology, Faculty of Fisheries, Kasetsart University, 50 Ngamwongwan Road, Ladyao, Chatuchark, Bangkok 10900, Thailand; ffistwr@ku.ac.th (T.R.); ffisntc@ku.ac.th (N.C.); 2 Department of Veterinary Medicine, College of Veterinary Medicine, National Chung Hsing University, Taichung 40227, Taiwan; kiwi0720@hotmail.com.tw (Y.C.); chiayuhsieh@nchu.edu.tw (C.-Y.H.); kevin4100043004@gmail.com (Y.-K.L.); 3 Department and Graduate Institute of Pharmacology, National Defense Medical Center, Taipei 11490, Taiwan

**Keywords:** anesthetics, animal welfare, MS-222, 2-phenoxyethanol, eugenol, tilapia

## Abstract

**Simple Summary:**

Fish studies often require anesthetic drugs to render the fish amenable for experimental handling and to secure animal welfare. However, the optimal dose is not always available. In this study, we determined the optimal does of three commonly used anesthetics, eugenol (EUG), tricaine methanesulfonate (MS-222), and 2-phenoxyethanol (2-PE), for induction of surgical anesthesia in marketable-size Nile tilapia, and decided on their minimum effective concentrations (MEC) in the fish serum. The results revealed that the optimal doses of EUG, MS-222, and 2-PE were 90, 300, and 900 ppm, and their MECs were 53, 70, and 263 µg/mL, respectively. Increasing the anesthetic doses generally resulted in the shortening of the induction times, but variably affected the recovery times. In contrast, the MECs were found to be independent of the administered doses. After the dosing was stopped, the serum concentrations of anesthetics decreased rapidly, lowering by >90% within the first hour and by >99% after 4 h. Our research provides practical information for a smooth fish handling and offered insights for designing researches requiring surgical anesthesia.

**Abstract:**

Anesthetic agents are often used in fish experiments to reduce the stress and struggle and to improve animal welfare. The present study aimed to determine the optimal doses and serum minimum effective concentration (MEC) of tricaine methanesulfonate (MS-222), 2-phenoxyethanol (2-PE), and eugenol (EUG) in Nile tilapia. Twenty-one fish were immersed in three different doses of each anesthetic and the minimal dose that produce stage III anesthesia within 5 min, maintain anesthesia status for 3 min, and recover within 5 min was considered the optimal dose. The serum concentrations of anesthetics immediately after the fish reached stage III anesthesia was defined as the MEC. The results revealed that the anesthetics dose-dependently shorten the induction time while the effect of doses on the recovery times were variable. The determined optimal doses for MS-222, 2-PE, and EUG were 300, 900, and 90 ppm, respectively. The MECs were 70, 263, and 53 µg/mL, respectively, about two to four times lower than the optimal doses and were independent of the doses. After immersion stopped, the serum concentrations decreased by >90% within the first hour and >99% after 4 h. Our research provides useful information for a smooth fish handling and design for researches requiring stage III anesthesia.

## 1. Introduction

To ensure animal welfare, anesthetic agents are commonly used to induce sedation and/or anesthesia in fish for many occasions such as during netting, weighing, handling, transportation, vaccination, blood sampling, surgery, and scientific researches [1,2]. Depending on the purpose of activity, various degrees of anesthesia stages are implemented. According to Coyle et al. [3], stages of anesthesia can be classified into four levels: stage I, sedation (characterized by reduction of motion and breathing), stage II, anesthesia (partial loss of equilibrium but still reactive to touch stimuli), stage III, surgical anesthesia (total loss of equilibrium and no reaction to touch stimuli), and stage IV, death (stop breathing and heartbeat). For invasive procedures such as surgery, drug injection, and blood sampling, in which tissue damages will occur, surgical anesthesia is advised [3,4]. 

The recommended doses of commonly used anesthetics for teleost fish are available in the literature but with considerable variations among fish species, and the information regarding the purpose of anesthetic uses was not totally known in those reports. In general, the recommended doses for tricaine methanesulfonate (MS-222) are 25–400 ppm, for 2-phenoxyethanol (2-PE) 0.25–1200 ppm, and for eugenol (EUG) 20–200 ppm [4,5,6]. Since conceivably the stage of anesthesia achieved may depend on the administered dose, the duration of exposure, the fish species, and the experimental conditions [1,3,7], the applied anesthetic dose should take these factors into account and no single universal dose is suitable for every fish species and condition. Consequently, to improve animal welfare, the optimal dose for a given anesthetic agent should be determined case-by-case in order to avoid inadequate anesthesia stage or overdosing.

Unlike antibacterial drugs, where the relationship between pharmacokinetic–pharmacodynamic (PK–PD) index and therapeutic outcome exists and permits the use of PK–PD modeling to determine the optimal dosage [8], the optimal dose of the anesthetic agent is conventionally determined by the dose titration approach in which the fish are exposed to different doses of anesthetics for varying periods of time. The dose that could provide an appropriate time frame for scientific procedure with rapid induction and recovery time would be considered optimal (see the Materials and Methods section).

Nile tilapia (*Oreochromis niloticus*) is one of the most popular farmed fish worldwide. They have been studied extensively as a target species with respect to fish nutrition, genetics, immunology, diseases, and many others. The anesthetic agents are frequently applied in the aforementioned studies to minimize the stress and struggle, rendering them amenable to handling during invasive procedures such as blood sampling or injection. The anesthetic doses specific for tilapia have been determined by sporadic studies, for example, 120 ppm for MS-222 [9], 385–2000 ppm for 2-PE [10,11], and 20–100 ppm for EUG [9,12]. Most of these studies investigated the effects of anesthetics in the small-sized tilapia, usually below 100 g, and the anesthetic doses suitable for relatively larger size tilapia have rarely been reported. It should be mentioned that many kinds of experiments involving surgery and the pharmacokinetic studies that are critical for the determination of optimal dosages and withdrawal time utilize adult or marketable-size fish. Similarly, the minimum effective concentration (MEC) of anesthetics in the fish serum is usually overlooked in most researches even though it can be a good circumstantial evidence supporting the rational use and proper dose of the drug. Therefore, the present study aimed to determine the optimal doses of the three commonly used anesthetic agents, namely, MS-222, 2-PE, and EUG in marketable-size Nile tilapia (about 500 g) as well as the serum MEC of each anesthetic. With the use of the optimal dose, the applied anesthetic could effectively produce proper anesthesia status while minimizing any potential adverse effects and thus improving animal welfare. The information of the serum MEC, which is frequently neglected in most fish anesthetic studies, would be a piece of circumstantial evidence supporting the optimal dose usage. Overall, the results of the current study would be beneficial for reasonable uses of these three common anesthetics in multi-purpose scientific researches in fish, especially the cichlid species, when these information are otherwise unavailable.

## 2. Materials and Methods 

### 2.1. Chemicals

Reference standards of MS-222 (analytical standard grade), EUG (99%), and sodium bicarbonate (NaHCO_3_) were purchased from Sigma-Aldrich (St. Louis, MO, USA). The 2-PE (99%) was from Acros Organics (Carlsbad, CA, USA). Acetonitrile (HPLC grade) and ethanol were from Avantor Performance Materials (Center Valley, PA, USA). Methanol (HPLC grade) was from Honeywell Burdick & Jackson (Muskegon, MI, USA). Sodium di-hydrogen phosphate anhydrous (NaH_2_PO_4_) and di-sodium hydrogen phosphate anhydrous (Na_2_HPO_4_) were from Panreac Química SLU (Barcelona, Spain).

### 2.2. Experimental Fish

A total of 21 clinically healthy Nile tilapia 400–600 g in weight were obtained from a commercial fish farm in Chiayi County, Taiwan, and were kept in an outdoor concrete pond at the College of Veterinary Medicine, National Chung Hsing University, Taiwan. Each individual fish was acclimatized in a 70 L tank containing freshwater for 5–7 days before drug administration. The water temperature throughout the study was controlled at 28 °C by a 120 W aquarium heater (Tzong Yang Aquarium, Tainan, Taiwan) in an air-conditioned room. Dissolved oxygen (DO) was maintained at ≥ 5.0 ppm, pH was in the range of 7.0–8.0, and the total ammonia nitrogen was <1.0 ppm. Temperature, DO, and pH were measured by a portable water quality meter (Lutron WA-2017SD, Lutron Electronics, Coopersburg, PA, USA). The animal study was approved by the Institutional Animal Care and Use Committee of National Chung Hsing University (IACUC approval No.109-048).

### 2.3. Preparation of Anesthetic Solutions

The solutions of MS-222 (150, 225, and 300 ppm) were prepared by dissolving the MS-222 reference standard in tap water buffered with sodium bicarbonate at a ratio of 2:1 (NaHCO_3_:MS-222) [13] to maintain the water pH of about 7. Because of the high solubility of 2-PE at the experimented concentrations (500, 700, and 900 ppm), the reference standard of 2-PE was dispersed directly into the distilled water. In contrast, EUG was initially dissolved in 99% ethanol at the ratio of EUG:ethanol of 1:9 [14] before being further diluted in the distilled water to obtain the predetermined concentrations (70, 80, and 90 ppm). Solutions of all anesthetic agents were freshly prepared before use. Note that the concentration unit of all anesthetic agents in this study was part per million (ppm) (mg/L).

### 2.4. Determination of the Optimal Doses Following Single Immersion Administration

For the purpose of the study, the optimal dose is defined as the minimal dose that could induce stage III anesthesia within 5 min, maintain stage III anesthesia for 3 min, and has a recovery time of less than 5 min after the fish is put back into the recovery tank [5,7]. These criteria are chosen based on the timeframe the anesthetic agent could provide for multiple scientific procedures and general activities. To determine the optimal anesthetic doses, the dose titration approach with a cross-over design was used. Specifically, a single fish would be sequentially exposed to the three different doses of the same anesthetic agent with a 1-week wash out period between any two consecutive doses. 

Twenty-one Nile tilapia were randomly distributed into one of the three groups, namely, MS-222, 2-PE, and EUG groups (*n* = 7 for each anesthetic). Taking the MS-222 group as an example, each fish was immersed with a given dose of MS-222 (150, 225, and 300 ppm) in a 10 L tank until stage III anesthesia was achieved (the induction phase). Then, the fish were monitored for another 3 min for the anesthesia status (the maintenance phase) before they were put back into a 70 L recovery tank (the recovery phase). The water temperature during the induction and recovery phases was controlled at 28 °C. Similarly, the doses of 500, 700, and 900 ppm 2-PE were tested on another 7 fish, and the doses of 70, 80, and 90 ppm EUG were tested, as well. The induction and recovery times of all groups were recorded. The fish that could not reach stage III anesthesia within a maximum observation period of 13 min were excluded from the statistical analysis. Note that the experimental doses were selected based on our preliminary results with 2 tilapia for each drug at their recommended doses of 150 ppm for MS-222, 500 ppm for 2-PE, and 60 for ppm EUG [3]. Higher doses were employed for subsequent testing because these preliminary doses failed to induce anesthesia within 3 min while some were successful within 5 min.

### 2.5. Determination of the MECs and the Serum Concentration-Time Profiles

Once the fish attained stage III anesthesia, they were removed from the water and the blood (0.1 mL) was collected from the caudal vessels using a 1 mL syringe with 22G needle without anticoagulant at 0, 0.25, 0.5, 1, 2, and 4 h. The blood samples were allowed to clot at room temperature and centrifuged at 3500 rpm (2191× *g*; KN-70, Kubota, Japan) for 10 min; the supernatants were collected and kept at −70 °C until analysis. The procedures of HPLC analysis are described below.

The serum concentration immediately after the fish reached stage III anesthesia (t = 0 h) was considered the MEC. To avoid confusion between the concentration of anesthetics in the water and those in the serum, the term ppm was used for the water concentration (anesthetic dose) while µg/mL was used for the serum concentration.

### 2.6. Sample Preparation and HPLC Analysis of Anesthetics in the Fish Serum

The serum samples (50 µL) were mixed with 150 μL of acetonitrile containing 0.1% formic acid in a 0.5 mL microcentrifuge tube and centrifuged at 3500 rpm (2191× *g*) for 10 min. The supernatant was transferred into a new 0.5 mL microcentrifuge tube and the acetonitrile with 0.1% formic acid was added to obtain a final volume of 200 μL. At the final step, 60 μL of the supernatant containing MS-222 was mixed with 140 μL of the phosphate buffer (10 mM NaH_2_PO_4_-Na_2_HPO_4_, pH 5). For the samples containing 2-PE and EUG, 68 and 90 μL of the supernatants were mixed with 132 and 110 μL of the phosphate buffer, respectively. All samples were filtered through 0.2 μm nylon syringe filters prior to the HPLC analyses.

The HPLC system consisted of a pump (1260 Infinity II, Agilent Technologies, Santa Clara, CA, USA), a UV-visible detector (1260 Infinity II, Agilent Technologies, Santa Clara, CA, USA), and a C-18 column sized 150 × 4.6 mm (Apollo, Hichrom, UK) for the MS-222 and 250 × 4.6 mm (Mightysil, Kanto, Japan) for the 2-PE and EUG analyses. The mobile phases were mixtures of acetonitrile, methanol, and the phosphate buffer (10 mM NaH_2_PO_4_-Na_2_HPO_4_, pH 5) at a ratio of 30:0:70, 17:17:66, and 45:0:55 *v*/*v* for the analysis of MS-222, 2-PE, and EUG, respectively. The detection wavelengths were 220, 218, and 200 nm; the flow rate was 1 mL/min and the injection volume was 50 μL. 

To establish the matrix calibration curves for quantification of the anesthetics, the reference standard was spiked into the blank fish serum at final concentrations of 100, 500 ng/mL, 1, 5, 10, and 50 μg/mL, then extracted and analyzed by the HPLC method described above. The weighting factor of 1/x^2^ was applied to improve the accuracy at the lower concentrations of the calibration curve. The limit of detection (LOD) and the limit of quantification (LOQ) were calculated by 3.3 × σ/S and 10 × σ/S, respectively (σ is the standard deviation of the y-intercept of the regression line, S is the slope of the calibration curve).

### 2.7. Statistical Analysis

Statistical analysis was performed using IBM SPSS Statistics version 25 software (IBM Corporation, Armonk, NY, USA). The differences of the induction times, recovery times, and MECs among the three doses were analyzed by one-way within-subjects (repeated measures) ANOVA. The differences were considered significant if *p*-value < 0.05.

## 3. Results

### 3.1. HPLC Method Validations for Quantification of Anesthetics 

**Figure 1:**
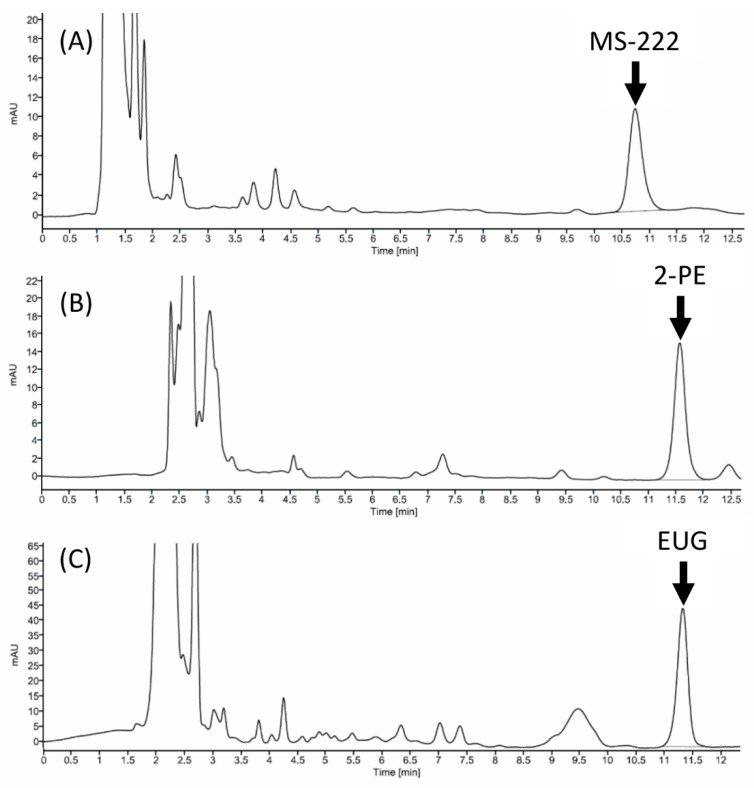
The representative chromatograms of 1 µg/mL reference standard of anesthetics in tilapia serum. The mobile phases consisted of acetonitrile, methanol, and the phosphate buffer (10 mM NaH2PO4-Na2HPO4, pH 5) at a ratio of: (**A**) 30:0:70 *v*/*v* for MS-222 (UV 200 nm); (**B**) 17:17:66 *v*/*v* for 2-PE (UV 218 nm); (**C**) 45:0:55 *v*/*v* for EUG (UV 200 nm).

The matrix calibration curves were linear over the range of 100 ng/mL to 50 μg/mL for all three anesthetics with the weighted r^2^ of 0.9968, 0.9938, and 0.9891 for MS-222, 2-PE, and EUG, respectively. The LODs were 153, 47, and 89 ng/mL, and the LOQs were 463, 143, and 269 ng/mL, respectively. The extraction recoveries were 88–108%, 100–110%, and 80–97%, respectively. The accuracies were 95.7–96.6%, 96.7–97.8%, and 96.6–98.5%, the intra-day precisions were 0.23–1.63%, 0.15–4.61%, and 0.22–2.70%, and the inter-day precisions were 0.78–1.91%, 0.45–3.01%, and 0.58–2.81%, respectively, for MS-222, 2-PE, and EUG. The retention times were 10.7, 11.5, and 11.4 min (Figure 1). 

### 3.2. Determination of the Optimal Doses Following Single Immersion Administration.

The selection of the optimal dose was based on the behavior response to the anesthetic agents mentioned above. All fish passed the criteria for recovery time (in less than 5 min) on the three doses (Table 1). However, only the highest dose of each anesthetic (i.e., MS-222 300 ppm, 2-PE 900 ppm, and EUG 90 ppm) fulfilled the criteria of induction time (less than 5 min) and maintaining stage III anesthesia for 3 min in all fish. Thus, the 300, 900, and 90 ppm were considered optimal doses for MS-222, 2-PE, and EUG, respectively.

During the induction phase, the fish showed sign of lower opercula ventilation rate, loss of equilibrium, and expected loss of reaction to touch stimuli. During the recovery process, the fish rapidly resumed normal opercula movement and consciousness as well as reactions to touch stimuli. Increasing the anesthetic doses resulted in the shortening of the induction time in a dose-dependent manner; however, the differences were statistically significant only in the MS-222 group (*p* < 0.05) (Table 1). In contrast, the effects of the doses on the recovery times were variable. Whereas the prolongation of the recovery time with increasing doses could be demonstrated in the MS-222 group (*p* < 0.05), no dose-dependency was observed in the other two anesthetic groups. 

### 3.3. Determination of the MECs and the Serum Concentration-Time Profiles.

For each anesthetic agent, despite different immersing dose levels, the MECs to reach stage III anesthesia were similar among anesthetics (Table 1). Because the MECs of the three doses were either not significantly different (MS-222 and 2-PE groups) or not showing dose-dependency (EUG group), the MECs from different doses were combined into a single grand mean and the MECs for MS-222, 2-PE, and EUG were 70.40 ± 22.06, 262.57 ± 90.33, and 53.41 ± 18.36 µg/mL, respectively. 

**Figure 2:**
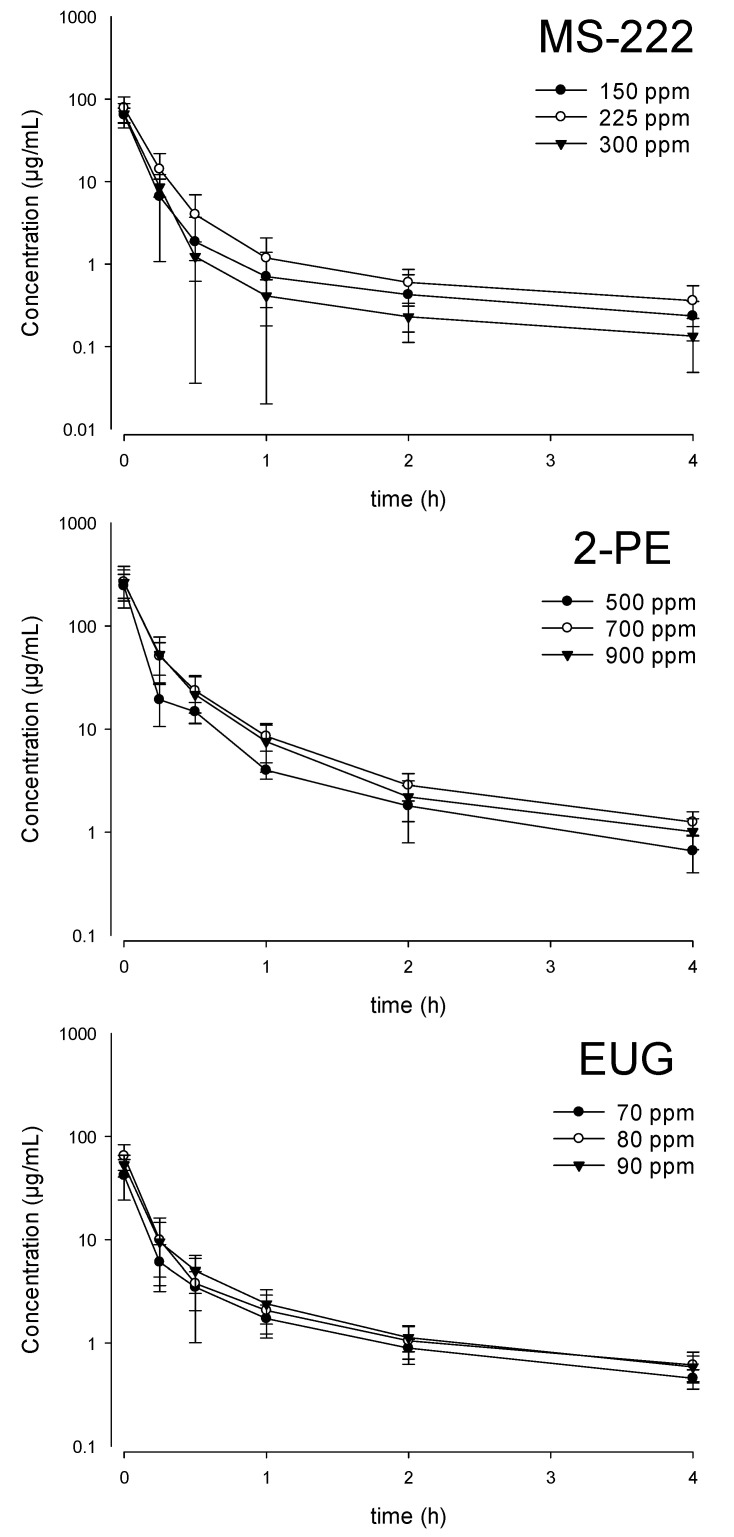
Serum concentration time profile (mean ± SD) of tricaine methanesulfonate (MS-222), 2-phenoxyethanol (2-PE), and eugenol (EUG) in Nile tilapia during the recovery phase following immersion at 3 different doses at 28 °C (*n* = 7).

**Figure 3:**
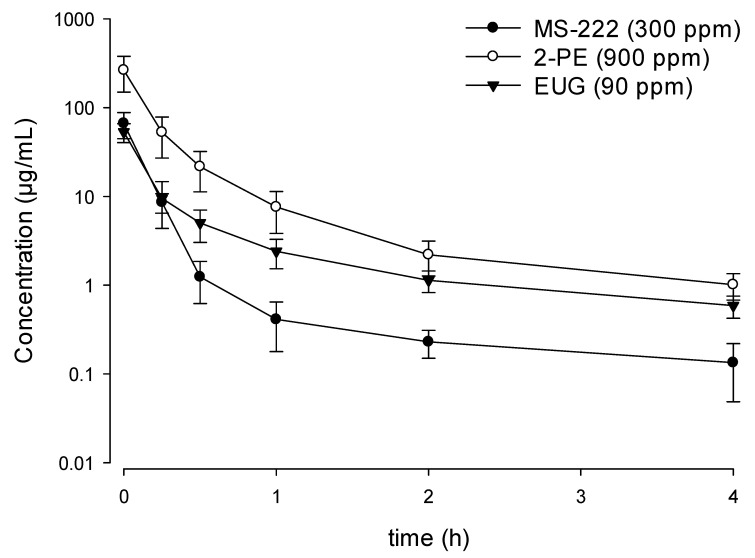
Serum concentration time profile (mean ± SD) of tricaine methanesulfonate (MS-222), 2-phenoxyethanol (2-PE), and eugenol (EUG) in Nile tilapia during the recovery phase following immersion at the optimal doses at 28 °C (*n* = 7).

The serum concentration time profiles of the three anesthetics at the three doses after the termination of the bath administration are presented in Figure 2. Following the transfer of the fish into the recovery tanks, the serum concentration of the three anesthetics revealed a multi-exponential decay with rapid elimination. All three anesthetics decreased by more than 90% within the first hour, and at the last blood sampling time point (t = 4 h), only 0.2–0.5% of MS-222 remained in the serum. Similarly, only 0.3–0.5 and 0.9–1.1% of 2-PE and EUG remained in the serum after 4 h (data not shown). At the optimal doses, the highest serum concentration was observed in the 900 ppm 2-PE groups, followed by the 90 ppm EUG and the 300 ppm MS-222 group (Figure 3); the serum concentrations at 4 h were 1.01, 0.59, and 0.13 μg/mL, respectively.

## 4. Discussion

“Can fish feel pain?” is an ongoing debate among researchers as many have claimed that fish do have sensation of pain while others disagree [15,16,17,18]. For the sake of animal welfare, it is wise to err on the safe side such that the use of anesthetics in fish experiments, especially for invasive procedures, is encouraged whenever applicable [1,2].

Similar to many drugs, the effectiveness of anesthetic agents in aquatic animals depends on several factors such as the fish species [19,20,21] and the rearing environment, especially the water temperature [19,22,23,24,25]. Therefore, in order to maximize a drug’s efficacy while minimizing its toxicity, an optimal dose for a given fish species in a specified environment is preferred. However, the definition of an optimal dose may vary depending on different research purposes. For example, most studies referred to the optimal dose as the lowest dose that could produce anesthesia with an induction time within 3 min and a recovery time within 5 min [7,21,26,27,28]. In contrast, other researchers may use an induction time of less than 3–5 min [20] or 5–10 min [5] and a recovery time of less than 10 min [19,20,25]. Our preliminary study revealed that Nile tilapia at this size could not be anesthetized within 3 min even though the maximum recommended doses were applied [3]. Consequently, we decided to use an induction time of less than 5 min as the criteria for the optimal dose, as opposed to the more commonly used 3 min. The general criterion of a 5-minute recovery time was chosen because Nile tilapia could quickly resume their response to stimuli after the cessation of anesthetics. Please refer to the Materials and Methods section for the method of choosing the optimal dose in the present study. Based on the aforementioned criteria, the determined optimal doses of the three anesthetics were generally higher than those found in the literature regardless of fish species, body weight, or experimental conditions. For instance, the optimal doses of MS-222 were reported to be 75 ppm for Senegalese sole [26], 100–125 ppm for marbled spinefoot [27], 120 ppm for Nile tilapia fry [9], 140 ppm for spotted sea bass [25], and 75–200 ppm for four tropical aquarium fishes [21]. Similarly, the optimal doses of 2-PE were 167 ppm for white sea bream and sharp snout sea bream [20], 300–350 ppm for European sea bass and 300–450 ppm for gilthead sea bream [19], 400 ppm for marbled spinefoot [27], and 600 ppm for Senegalese sole [26]. The optimal doses of EUG were 20 ppm for Nile tilapia fry [9], 50 ppm for red tilapia juvenile [12], 60 ppm for spotted sea bass [25], and 80 ppm for freshwater angelfish [28]. The reason why the current results are almost always greater than the other studies might be mainly due to the species differences, as tilapia usually requires higher anesthetic doses when compared to other fishes [3,6,7]. The physiological characteristics responsible for the mechanism are not well understood, but might be related to the greater intrinsic capability of tilapia to metabolize and/or excrete xenobiotics and being a warm-water fish that in general metabolically more active than the cold-water fish species [22,23]. The influence of body weight on the anesthetic effects is even less clear. It is interesting to note that the marketable-size tilapia (from the current study) required higher doses for anesthetic induction when compared to the fry or juvenile tilapia [9,10,11,12]. Whether or not the small-sized fish has faster absorption and/or lower MEC than the bigger fish remain to be elucidated. Since the drug effect is also dependent on the experimental conditions and environmental factors, direct comparison and interpretations among different studies should be performed with caution [20,22,23,24,26]. Among the anesthetics evaluated, EUG showed the lowest effective concentration while 2-PE was the highest. The EUG worked at a concentration three times lower than MS-222 and 10 times lower than 2-PE. A similar phenomenon of smaller effective EUG concentrations has been reported in several other species, with doses about two to six times smaller than MS-222 in spotted sea bass [25], hickory shad [29], and Nile tilapia [9], and doses 4 to 20 times smaller than 2-PE in hickory shad [29], shabout [30], and European sea bass [31]. From the economic aspect, although EUG is more expensive than 2-PE calculated by weight, the EUG is the cheapest of the three anesthetics used in the present study calculated by every 10 L of each anesthetic at optimal concentrations (USD 20 for MS-222, USD 0.8 for 2-PE, and USD 0.6 for EUG, based on the price from the sources listed in the Materials and Methods section). With the lowest effective dosage, EUG not only was the most economic choice, but also the one that produces lowest pollution by weight for the environment, making it a desirable choice from a practical viewpoint.

The decrease in the induction time as the anesthetic dose increases could be reasonably expected. At a higher dose, the drug reaches the threshold concentration (the MEC) earlier, and thus produces the pharmacological action (anesthesia) sooner. In fact, this is one of the most consistent outcomes in studies with different anesthetic doses, including the three agents used [9,11,27,29,32]. The lack of statistically significant differences in the induction times at different dosages of the EUG and 2-PE groups in the present study was likely due to the high variation in the individual fish response and the relatively small differences between the experimental dose levels to show statistically different outcomes. Nevertheless, the trends of dose-dependent shortening in the induction times of the two drugs could still be acknowledged. On the other hand, the effects of increasing doses on the recovery time were more variable. Some studies found more sensible direct relationships (increasing the dose prolongs the recovery time) for MS-222 in Nile tilapia [the current study], Nile tilapia fry [9], goldfish [32], zebrafish, guppy, discus [21], and spotted sea bass [25]; for 2-PE in hybrid tilapia juvenile [11], European sea bass [31], goldfish [32], and hickory shad [29]; and for EUG in Nile tilapia fry [9], red tilapia juvenile [12], and spotted sea bass [25]; while other studies, albeit less common, reported the reverse trend (increasing the dose leading to the shortening of the recovery time). This seemingly counterintuitive outcome is attributed to the fact that the duration of the anesthetic exposure is usually also be reduced at a higher dose (thus a shortened induction time) such that lower drug uptake and faster removal is expected [19]. The specific examples include MS-222 in common carp [33], Senegalese sole [26], green swordtail [21], and hickory shad [29]. On the same note, there were studies that found no clear correlation between the anesthetic doses and the recovery time; specific examples also include MS-222 [27,34], 2-PE [the current study and 19,26,27], and EUG [the current study and 28,29,31]. The absence of significant differences in the recovery times at different dosages of the EUG and 2-PE groups in the present study likely reflected the real lack of significance at the tested doses as the dose-dependent responses were not observed. This phenomenon might be understandable from the pharmacokinetic principle assuming the first-order kinetics of drug elimination: the higher the dose, the faster the elimination rate [35]. Consequently, the elimination half-life of the drug did not change significantly with dose (dose-independent), nor did the recovery time. Overall, the effect of anesthetic doses on the recovery time depends on the interplay between the pharmacokinetic and pharmacodynamic properties of a drug, which might also be confounded by experimental and environmental conditions. It is not clear which physiological and biochemical differences characteristic to each fish species contributed to the various relationship between the doses and the recovery time, but this is certainly worth further investigation. 

The fact that fish immersed in higher doses required shorter exposure time to reach stage III anesthesia most likely explained the similar MECs across the three different doses regardless of anesthetics. Despite the high inter-individual variability, the average MECs of MS-222 (70 µg/mL) and EUG (53 µg/mL) were lower than that of the 2-PE (263 µg/mL), suggesting lesser potency of 2-PE in inducing stage III anesthesia in Nile tilapia. When MECs of different fish species were compared, the MEC of MS-222 in Nile tilapia was four times higher than that in the Atlantic salmon (70 vs. 16 µg/mL) [36] and 24 times higher (53 vs. 2.2 µg/mL) than the MEC of EUG in the Japanese flounder [37]. These findings were in agreement with the general need for higher optimal doses in Nile tilapia compared to other fish species, as previously mentioned [3,6,7]. These data once more highlight the necessity of obtaining specific information beforehand on the choice of anesthetic and its optimal dose for proper handling of the fish and getting trustful research results.

Following the transfer of the Nile tilapia to the recovery tanks, the serum concentrations of the three anesthetic agents declined rapidly (and multi-exponentially) with a similar trend. To date, there is no information available for the serum/plasma half-lives of these three agents in Nile tilapia. Unfortunately, based on the nature of the drug (an anesthetic agent) and the limited 4 h sampling time, we could not reasonably assure whether the sampling length surpassed the distribution phases and reached the elimination phases long enough, and therefore the half-lives were not calculated or reported in the current study. The published serum/plasma half-lives of MS-222, which were 1.7 min (distribution half-life) in the Atlantic salmon [36] and 56 min in the spiny dogfish [38], were found to be much shorter than the half-lives of EUG, which were 12.1 h in the rainbow trout [39] and 19.8 h in the grass carp [40], whereas the serum/plasma half-life of 2-PE in fish has not been reported thus far. In contrast, the muscle half-lives of MS-222, 2-PE, and EUG in tilapia were less different and reported to be 53.5, 147.8, and 52.5 min, respectively [41]. While the current design prevented us from providing confident estimation of serum half-lives and other PK parameters, it appeared that based on the percentage of the remaining drug at the last time point (t = 4 h) relative to the initial concentration (t = 0 h), the elimination of MS-222 at the optimal dose was very rapid and more rapid than the EUG and 2-PE. Although the elimination half-lives of the three anesthetics could not be accurately calculated (as mentioned above) due to the lack of anesthetic half-life information in this fish, we provisionally derived the half-lives from the slopes of the concentration-time curve based on the last three time points, and the estimated half-lives for MS-222, 2-PE, and EUG in Nile tilapia at their optimal doses were all around 1.5 h (data not shown). This information could serve as the first estimation of the time scale of the half-lives of these commonly used anesthetic agents. The apparently shorter terminal half-lives of anesthetic drugs in Nile tilapia compare to other teleosts [39,40] was in agreement with the higher optimal doses re-quired to attain the stage III anesthesia status and might be related to the higher met-abolic rate of this warm-water species. It should also be mentioned that regarding the effect of the rearing temperature, increase water temperature generally accelerate both induction time and recovery time, no matter in cold-water fish such as Atlantic cod [22], warm-water fish such as marbled rabbitfish [24], or eurythermal fish such as Eu-ropean sea bass and gilthead sea bream [19]. However, this conclusion could not be applied in every situation as Atlantic halibut showed longer recovery time in warmer water [23] and spotted sea bass even exhibited dose-dependent temperature effects [25]. The influence of water temperature on anesthetic action is possibly species- and drug-specific such that the general assumption of faster induction and recovery pro-cesses at higher temperature should be treated with caution.

In short, the current study provides the optimal doses of MS-222, 2-PE, and EUG for multi-purpose uses in Nile tilapia experimented at 28 °C that require stage III anesthesia such as surgery, blood collection, and various routes of drug administrations. The results also indicated that tilapia often require higher anesthetic doses compared to other fish species, which was likely related to the higher MECs required to attain the same level of anesthesia. The results could possibly be representative for fishes of various genus levels in which such information were rare or otherwise unavailable.

## 5. Conclusions

The optimal doses to induce stage III anesthesia within 5 min, maintain anesthesia status for 3 min and recover within 5 min, were determined for the first time for MS-222 (300 ppm), 2-PE (900 ppm), and EUG (90 ppm) in marketable-size (about 500 g) Nile tilapia cultured at 28 °C. The rarely reported MECs (70, 263, and 53 µg/mL, respectively) were two to four times lower than the optimal doses and were found to be independent of the anesthetic doses, a novel discovery. The serum elimination half-lives of the three anesthetics at their optimal doses were provisionally provided. The current study highlighted the benefits of obtaining information specific to the anesthetic and its optimal dose beforehand. The effect of environmental factors such as temperature and salinity on the optimal doses and MECs of anesthetic agents is worth future investigation.

## Figures and Tables

**Table 1:** The induction time, recovery time, and minimum effective concentration (MEC) of tricaine methanesulfonate (MS-222), 2-phenoxyethanol (2-PE), and eugenol (EUG) in Nile tilapia at 28 °C following single immersion administrations (*n* = 7) ^A^.

Anesthetic Drug	Dose (ppm)	Induction Time (s)	Number of Fish That Had Induction Time < 5 min	Number of Fish That Could Be Maintained in Stage III Anesthesia for 3 min	Recovery Time (s)	MEC (µg/mL) ^B^	Grand Mean of MEC (µg/mL) ^B^
MS-222	150 ^C^	488.60 ± 125.38 ^a^	2/7	4/7	14.80 ± 15.01 ^a^	64.74 ± 12.84 ^a^	70.40 ± 22.06
	225	407.57 ± 173.19 ^a^^b^	5/7	6/7	23.00 ± 10.68 ^a^	78.48 ± 27.58 ^a^	
	300	153.43 ± 48.38 ^b^	7/7	7/7	47.00 ± 11.00 ^b^	66.36 ± 21.62 ^a^	
2-PE	500 ^D^	547.33 ± 173.02 ^a^	0/7	3/7	81.57 ± 54.06 ^a^	245.87 ± 71.24 ^a^	262.57 ± 90.33
	700	382.57 ± 57.52 ^a^	0/7	7/7	132.57 ± 45.94 ^a^	267.85 ± 82.21 ^a^	
	900	256.00 ± 47.72 ^a^	7/7	7/7	113.57 ± 32.58 ^a^	264.46 ± 114.59 ^a^	
EUG	70	236.71 ± 78.25 ^a^	4/7	7/7	60.57 ± 19.54 ^a^	41.97 ± 17.82 ^a^	53.41 ± 18.36
	80	223.00 ± 68.91 ^a^	5/7	7/7	61.71 ± 25.74 ^a^	65.07 ± 18.23 ^b^	
	90	164.71 ± 21.58 ^a^	7/7	7/7	54.14 ± 19.57 ^a^	53.19 ± 12.74 ^ab^	

^A^ The data are presented as mean ± SD. For a given anesthetic agent, means with different superscripts (lowercase letters) across columns are significantly different from each other (*p* < 0.05). ^B^ Minimum effective concentration (MEC) is defined as the serum anesthetic concentration immediately after the fish reached stage III anesthesia (t = 0 h). ^C^ The average values of 5 fish. The other 2 fish could not reach stage III anesthesia despite being exposed to MS-222 up to 13 min. ^D^ The average values of 3 fish. The other 4 fish could not reach stage III anesthesia despite being exposed to 2-PE up to 13 min.

## Data Availability

The data presented in this study are available on request from the corresponding author.
